# Computational Chemistry Study of pH-Responsive Fluorescent Probes and Development of Supporting Software

**DOI:** 10.3390/molecules30020273

**Published:** 2025-01-12

**Authors:** Ximeng Zhu, Yongchun Wei, Xiaogang Liu

**Affiliations:** School of Materials and Environment, Beijing Institute of Technology, Zhuhai 519088, China; zhuximeng.academic@gmail.com (X.Z.); wei_yc@bitzh.edu.cn (Y.W.)

**Keywords:** fluorescent probe, pH responsiveness, molecular dynamics simulation, quantum chemical calculation

## Abstract

This study employs quantum chemical computational methods to predict the spectroscopic properties of fluorescent probes 2,6-bis(2-benzimidazolyl)pyridine (BBP) and (*E*)-3-(2-(1*H*-benzo[*d*]imidazol-2-yl)vinyl)-9-(2-(2-methoxyethoxy)ethyl)-9*H*-carbazole (BIMC). Using time-dependent density functional theory (TDDFT), we successfully predicted the fluorescence emission wavelengths of BBP under various protonation states, achieving an average deviation of 6.0% from experimental excitation energies. Molecular dynamics simulations elucidated the microscopic mechanism underlying BBP’s fluorescence quenching under acidic conditions. The spectroscopic predictions for BIMC were performed using the STEOM-DLPNO-CCSD method, yielding an average deviation of merely 0.57% from experimental values. Based on Einstein’s spontaneous emission formula and empirical internal conversion rate formulas, we calculated fluorescence quantum yields for spectral intensity calibration, enabling the accurate prediction of experimental spectra. To streamline the computational workflow, we developed and open-sourced the EasySpecCalc software v0.0.1 on GitHub, aiming to facilitate the design and development of fluorescent probes.

## 1. Introduction

Fluorescent probes have emerged as powerful tools in biomedical research and clinical applications, particularly in cancer diagnosis and treatment [1]. These molecular sensors, typically composed of fluorophores, spacers, and receptors, enable real-time visualization of biological processes at cellular and molecular levels [2]. During tumor growth, proliferation, and metastasis, cancer cells exhibit distinctive metabolic characteristics that significantly differ from normal cells. Extensive research has demonstrated that tumor cells display notable molecular expression abnormalities and unique microenvironmental features compared to normal cells. These abnormally expressed substances include cyclooxygenase-2 (COX-2), nitroreductase (NTR), glutathione (GSH), nicotinamide adenine dinucleotide (NADH), biological thiols, H_2_S, H_2_O_2_, and HOCl. Additionally, one of the characteristics of the tumor microenvironment is abnormal pH [3]. These biomarkers and microenvironmental features provide reliable indicators for tumor localization and grading. This understanding has facilitated the development of fluorescent probes with high sensitivity, specificity, and photostability, significantly enhancing the precision of tumor imaging and surgical navigation, thus offering new possibilities for early cancer detection and precise tumor resection [4]. Fluorescent probes have garnered considerable attention due to their excellent tissue penetration capability, minimal background interference, and negligible photodamage characteristics [5,6]. Furthermore, some fluorescent probes demonstrate dual functionality, combining imaging capabilities with therapeutic effects such as photodynamic or photothermal therapy, thereby pioneering new approaches for integrated cancer theranostics [7].

In recent years, despite significant advances in the development of tumor fluorescent probes, numerous challenges remain in predicting fluorescent spectra and elucidating their underlying mechanisms. While traditional chemical synthesis and experimental determination methods have proven feasible, their predictive capabilities and guiding value at the molecular design stage are still insufficient. To achieve specific design objectives, researchers typically need to undergo multiple rounds of chemical modifications to adjust fluorescent emission wavelengths, which severely constrains the development efficiency of tumor fluorescent probes. Recent studies have also investigated dyes and fluorescent probes using computational chemistry methods, such as predicting the excited-state properties of multi-resonance and thermally activated delayed fluorescence (MR-TADF) materials [8] and calculating BODIPY dyes [9]. These studies have demonstrated the potential of quantum chemical methods in predicting fluorescent spectra, though their practical applications remain limited. Therefore, it is crucial to explore and develop computational methods that enable researchers to calculate fluorescent spectra more simply and cost-effectively. These advances will deepen our understanding of fluorescent mechanisms and promote the precise design and efficient development of functionally controllable tumor fluorescent probes.

Time-dependent density functional theory (TDDFT) and STEOM-DLPNO-CCSD methods have demonstrated exceptional performance in predicting fluorescent spectra. These methods have shown high accuracy in reproducing experimental results across a wide range of molecular systems. In this study, we applied quantum chemical computational methods to predict and analyze the fluorescent spectra of potential fluorescent probes. We used TDDFT to predict the fluorescent spectrum of 2,6-bis(2-benzimidazolyl)pyridine (BBP), a fluorophore molecule with the same potential as a pH-regulated probe. Additionally, molecular dynamics simulations were employed to reveal its fluorescence quenching mechanism under acidic conditions. We subsequently combined TDDFT and STEOM-DLPNO-CCSD methods to predict the spectral properties of the established fluorescent probe *(E)*-3-(2-(1*H*-benzo[*d*]imidazol-2-yl)vinyl)-9-(2-(2-methoxyethoxy)ethyl)-9*H*-carbazole (BIMC) [10] and elucidated its pH-responsive mechanism through electrostatic potential and average local ionization energy analyses. To facilitate the application of quantum chemical calculations in fluorescent probe development, we developed the EasySpecCalc software with a graphical user interface, achieving an automated computational workflow for fluorescent spectra prediction.

## 2. Results and Discussion

### 2.1. BBP

**Figure 1:**
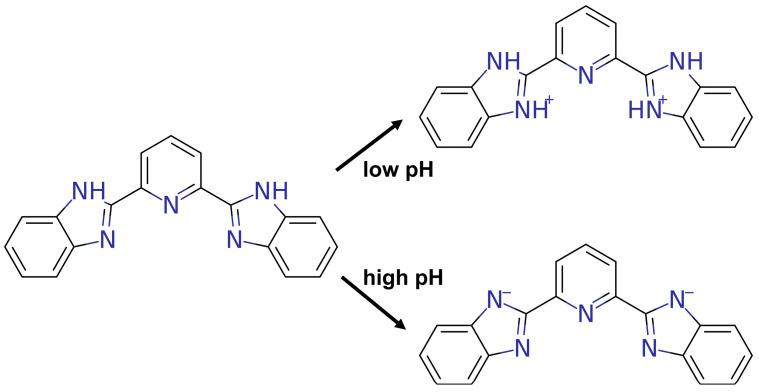
Structural representation of BBP and its pH-responsive forms.

**Figure 2:**
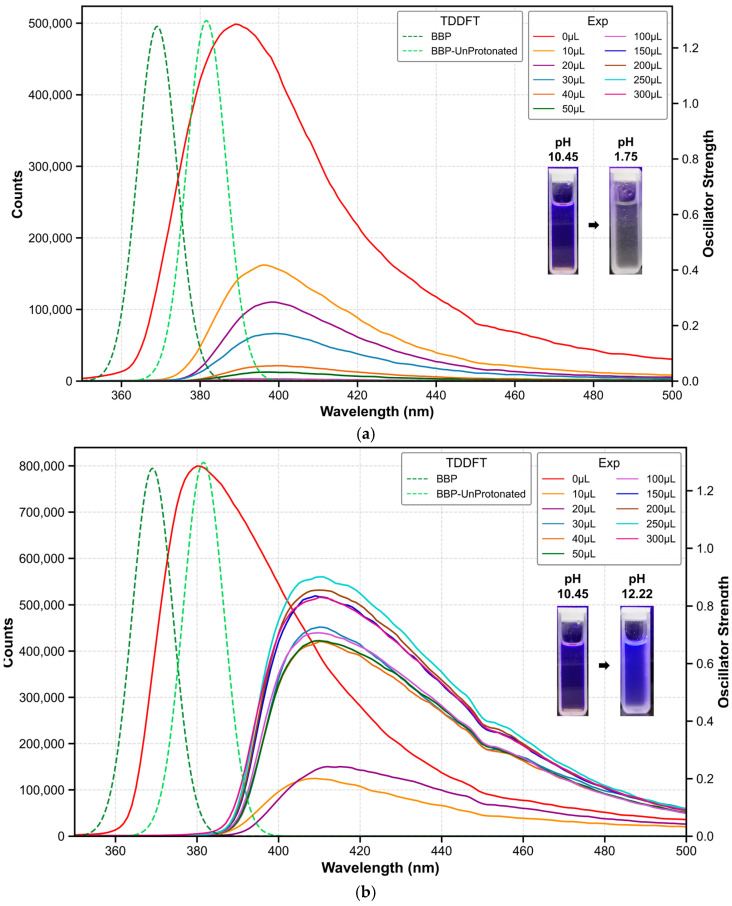
(**a**) Fluorescence emission spectra of BBP as a function of acid titration volume (0–300 μL), demonstrating pH-dependent spectral changes. The systematic pH decrease from initial 10.45 (0 μL) through intermediate values (9.57 at 10 μL, 3.85 at 20 μL, 3.30 at 30 μL, 3.25 at 40 μL, 3.17 at 50 μL, 2.54 at 100 μL, 2.20 at 150 μL, 2.08 at 200 μL, 1.80 at 250 μL, and finally 1.75 at 300 μL) induces a redshift of the maximum emission from 385 nm to 410 nm, accompanied by significant fluorescence quenching. Experimental data (solid lines) correspond to the left y-axis (counts), while TDDFT calculations (dashed lines) correspond to the right y-axis (oscillator strength). (**b**) Fluorescence emission spectra of BBP as a function of base titration volume (0–300 μL). The spectra demonstrate pH-dependent changes as the pH increases from the initial 10.45 (0 μL) through successive stages (10.86 at 10 μL, 11.05 at 20 μL, 11.51 at 30 μL, 11.67 at 40 μL, 11.87 at 50 μL, 11.92 at 100 μL, 12.00 at 150 μL, 12.04 at 200 μL, 12.14 at 250 μL, and finally 12.22 at 300 μL), showing an initial redshift of the maximum emission from 385 nm to 420 nm. During titration, the fluorescence first undergoes significant quenching, followed by gradual recovery. Experimental data (solid lines) correspond to the left y-axis (counts), while TDDFT calculations (dashed lines) correspond to the right y-axis (oscillator strength).

As shown in Figure 1, we constructed the structures of BBP and its protonated (BBP-Protonated) and deprotonated (BBP-Deprotonated) forms to investigate the conformational and spectral characteristics of BBP under different pH conditions. The predicted most basic pK_a_ was 4.63 (95% CI: 4.06–5.20), and the most acidic pK_a_ was 11.76 (95% CI: 10.78–12.74). Subsequently, conformational optimization was performed for all three configurations. The lowest energy conformers of each structure were obtained using the PM6-DH+ [11] method. Based on the optimized configurations, we employed the r2SCAN-3c [12] method to optimize the ground state (S0-min) structures. The electronic spectra were then studied using the PBE0 hybrid functional [13] with the def2-SV(P) basis set [14]. To validate the computational results, we conducted acid-base titration experiments to obtain the fluorescence spectra of BBP under various pH conditions. The theoretical calculations and experimental maximum emission wavelength data are summarized in Table 1. The acidic and basic response spectra of BBP are shown in Figure 2a and Figure 2b, respectively. The theoretically calculated maximum emission wavelengths for BBP, BBP-Protonated, and BBP-Deprotonated were 369.1, 372.3, and 381.6 nm, respectively, while the corresponding experimental values were 385.0, 396.0, and 410.0 nm, with excitation energies being overestimated by an average of 6.0%. This systematic deviation is acceptable in quantum chemical calculations, and the theoretical calculations successfully predicted the increase in emission wavelengths across different protonation states of BBP.

**Figure 3:**
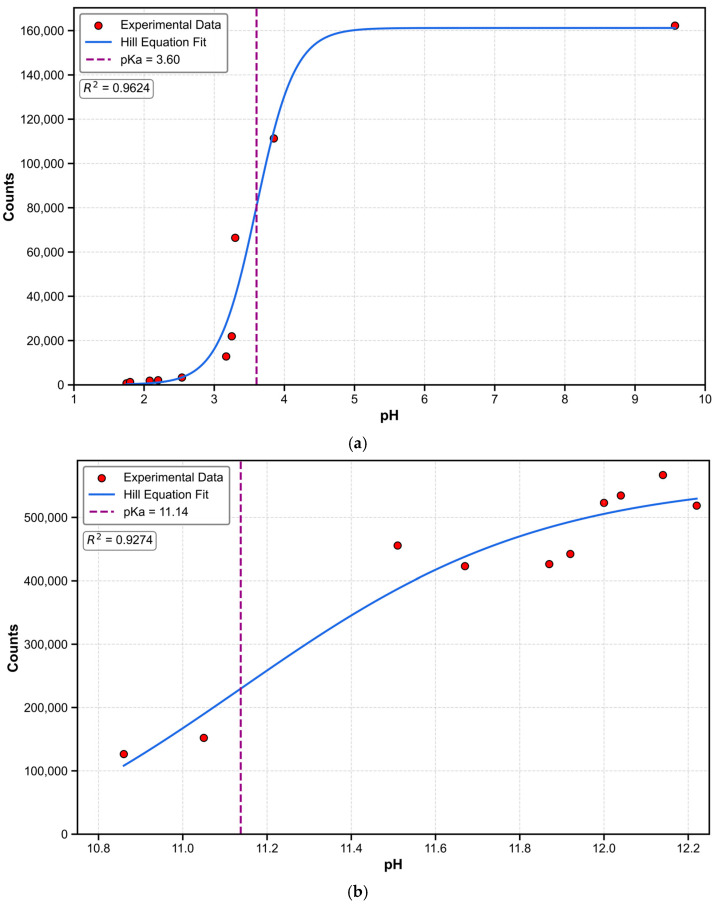
(**a**) Relationship between fluorescence intensity and corresponding pH for the BBP molecule under acidic conditions. Data points represent experimentally measured fluorescence intensity with corresponding pH values, and the curve represents the fit using the Hill equation, yielding the most basic pK_a_ value of 3.60, with a goodness-of-fit R^2^ of 0.96. (**b**) Relationship between fluorescence intensity and corresponding pH for the BBP molecule under basic conditions. Data points represent experimentally measured fluorescence intensity with corresponding pH values, and the curve represents the fit using the Hill equation, yielding the most acidic pK_a_ value of 11.14, with a goodness-of-fit R^2^ of 0.93.

The relationship between fluorescence intensity and pH for the BBP molecule was experimentally measured, and the data were fitted using the Hill equation to determine the pK_a_ values under acidic and basic conditions. During acidic titration, the Hill equation fitting showed the most basic pK_a_ value of 3.60, with a goodness-of-fit R^2^ of 0.96 (Figure 3a). During basic titration, the fitting revealed the most acidic pK_a_ value of 11.14, with a goodness-of-fit R^2^ of 0.93 (Figure 3b). These results are consistent with the predicted pK_a_ values. These findings demonstrate that the BBP molecule exhibits two distinct dissociation characteristics, corresponding to different pH ranges. This dual pK_a_ behavior is closely related to the presence of multiple acid-base active sites in the BBP structure.

**Figure 4:**
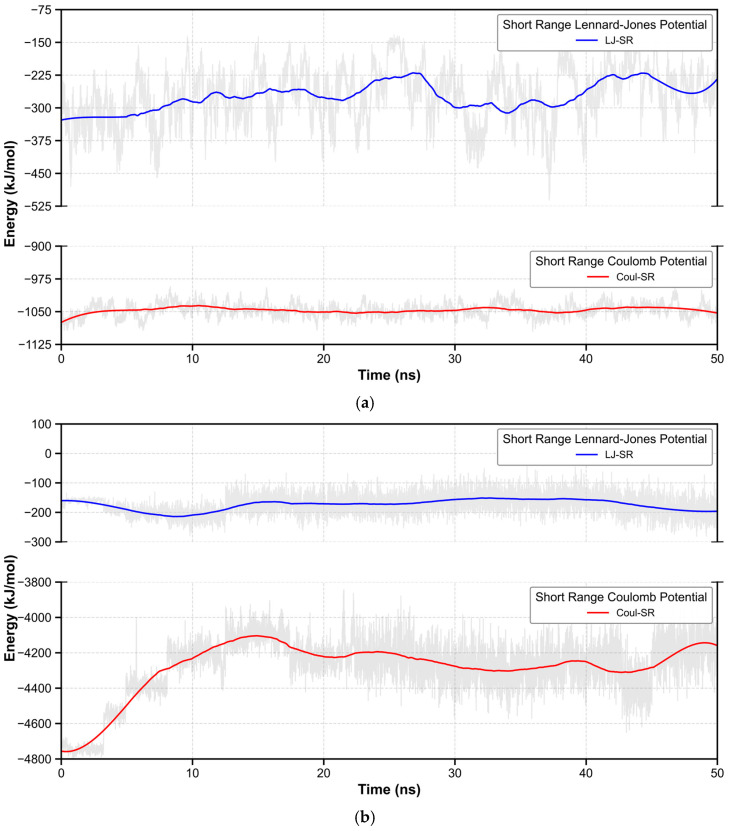
(**a**) Intermolecular interactions of BBP. (**b**) Intermolecular interactions of BBP-Deprotonated.

To further investigate the fluorescence quenching phenomenon observed under acidic conditions, we conducted molecular dynamics (MD) simulation studies. Through 50 ns MD simulations of BBP and BBP-Deprotonated in the DMSO solvent environment, we obtained information about the intermolecular interactions. As shown in Figure 4a, for the BBP, the average short-range Coulomb potential (Coul-SR) of intermolecular interactions was −1046.65 kJ/mol (RMSD = 15.59 kJ/mol; Tot drift = 4.38 kJ/mol), and the average short-range Lennard–Jones potential (LJ-SR) was −273.83 kJ/mol (RMSD = 62.82 kJ/mol; Tot drift = 58.79 kJ/mol). In contrast, as shown in Figure 4b, the BBP-Deprotonated system exhibited significantly enhanced intermolecular attractions. Its Coul-SR reached −4280.23 kJ/mol (RMSD = 170.97 kJ/mol; Tot drift = 215.17 kJ/mol), while the LJ-SR was −174.18 kJ/mol (RMSD = 32.68 kJ/mol; Tot drift = 16.84 kJ/mol). This marked difference in electrostatic interactions reveals that the deprotonation of BBP significantly altered the pattern of intermolecular interactions.

In the analysis of MD trajectories, we conducted a detailed investigation of dimer formation. Under strict criteria for dimer definition (an intermolecular distance threshold of 0.7 nm and a duration threshold of 0.5 ns), BBP-Deprotonated exhibited stronger dimerization tendency: 26 dimer events were observed with a mean lifetime of 1.403 ns (SD = 1.275 ns), while BBP showed only 7 dimer events with a mean lifetime of 0.714 ns (SD = 0.214 ns). When the intermolecular distance threshold was increased to 3 nm, 450 and 502 dimer events were observed for BBP-Deprotonated and BBP, respectively, with mean lifetimes of 1.251 ns (SD = 1.226 ns) and 1.082 ns (SD = 0.730 ns). The results demonstrate that BBP-Deprotonated forms more compact and stable dimers. BBP-Deprotonated preferentially forms dimeric structures with specific binding patterns, attributed to the charge distribution changes induced by deprotonation. Under acidic conditions, this enhanced intermolecular interaction and stable dimer formation provide additional non-radiative relaxation pathways, providing a molecular-level explanation for the detected fluorescence quenching phenomenon, while elucidating the photophysical behavior of BBP under different pH conditions.

### 2.2. BIMC

**Figure 5:**
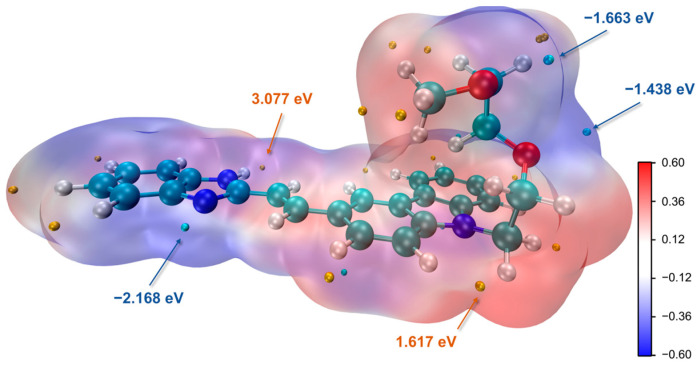
ESP maps of BIMC. The electrostatic potential was calculated on the surface with an electron density of 0.001 a.u., and the grid spacing was set to 0.25 Bohr. C: Cyan, N: blue, O: red, and H: white.

**Figure 6:**
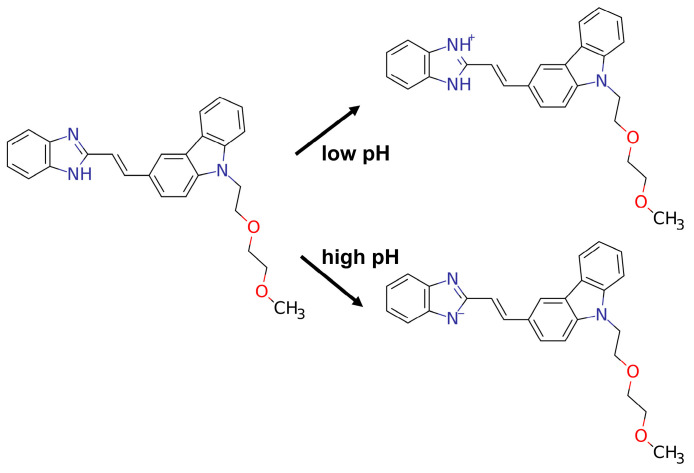
Structural representation of BIMC and its pH-responsive forms.

We determined the lowest energy conformation of the BIMC at the PM6-DH+ level. Subsequently, we optimized the ground-state (S0-min) structure using the r2SCAN-3c method, incorporating water as the solvent environment. To ensure that the obtained structure represented a local minimum, we performed frequency calculations to verify the absence of imaginary frequencies. Based on the optimized configuration analysis, we obtained crucial information comprising electrostatic potential (ESP) maps [15,16] and average local ionization energy (ALIE) [17,18], which provided insights into the molecular reactivity. The ESP map serves as an established tool to predict electrophilic and nucleophilic reaction sites, describing the interaction energy between a unit positive charge located at a point and the current system. ALIE characterizes the reactivity of atoms. Figure 5 shows the ESP distribution map. We investigated the reactivity characteristics of the benzimidazole group. The amino nitrogen atom exhibited a high electrostatic potential (+3.077 eV), indicating low electron density and a significant positive charge at this site. This positive charge suggests high affinity for nucleophilic reagents, making it a potential active center for nucleophilic attack. In contrast, the double-bonded nitrogen atom showed a lower electrostatic potential (−2.168 eV), reflecting high electron density and susceptibility to electrophilic attack. Under acidic conditions, this double-bonded nitrogen readily undergoes protonation to form an amino group. The ALIE distribution showed a minimum value of 7.694 eV near the double-bonded nitrogen and a maximum value of 14.025 eV at the amino nitrogen site. Based on theoretical calculations, we elucidated the corresponding behavior of BIMC in acidic and basic environments, as shown in Figure 6. The most basic pK_a_ that was predicted was 6.15 (95% CI: 5.50–6.80), and the most acidic pK_a_ was 12.98 (95% CI: 11.87–14.09). These observations align with chemical intuition and are supported by ^1^H NMR titration results [10].

**Figure 7:**
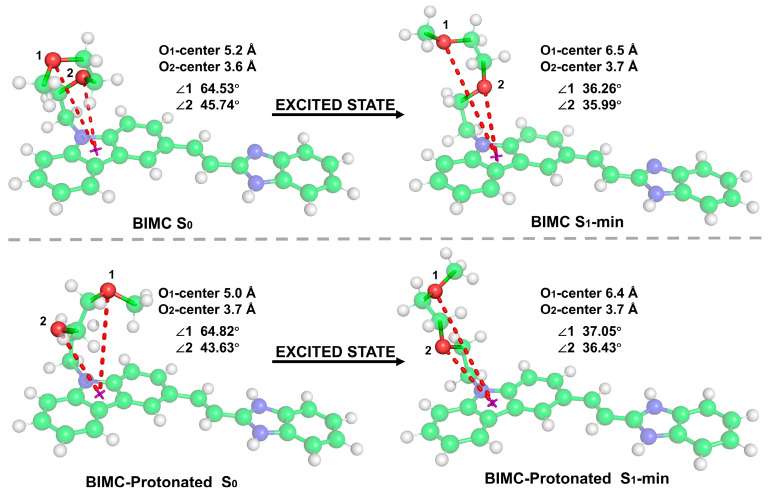
Optimized geometric structures of BIMC (upper panel) and BIMC-Protonated (lower panel) in the ground state (S0) and excited state (S1-min). The red dashed lines indicate the distances from oxygen atoms (O1 and O2) to the imidazole ring center. C: green, N: blue, O: red, and H: white.

**Figure 8:**
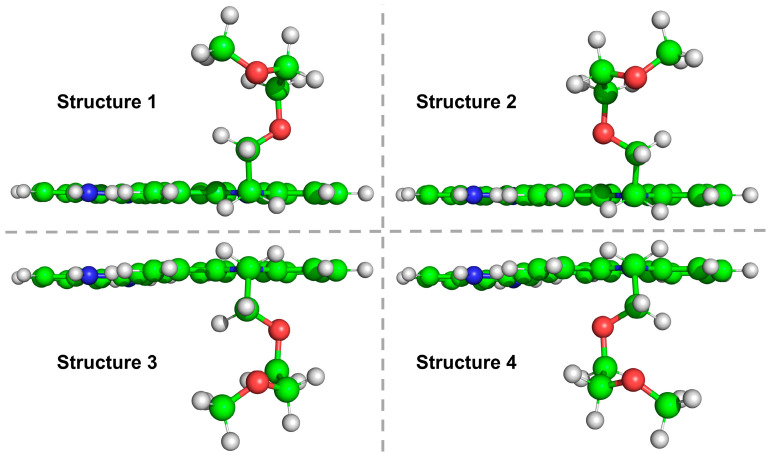
Four different conformational isomers (structures 1–4) of BIMC-Protonated in the S1-min state. C: green, N: blue, O: red, and H: white.

The electronic spectra and excited-state geometries of BIMC and its protonated form (BIMC-Protonated) in acidic environments were investigated using the PBE0 hybrid functional and def2-SV(P) basis set, accompanied by frequency calculations to verify the absence of imaginary frequencies. Figure 7 presents the optimized geometric structures of BIMC/BIMC-Protonated in both the ground state (S0) and excited state (S1-min). Results indicate that while the planar portions of BIMC/BIMC-Protonated showed no significant geometric changes in both S0 and S1-min states, the side chain attached to the imidazole ring underwent substantial conformational extension during the S0-to-S1 transition. Consequently, the two oxygen atoms on the side chain were numbered, and their distances to the imidazole ring center and corresponding angles were marked. In the S0 state of BIMC, the distances from O_1_ and O_2_ to the imidazole ring center were 5.2 Å and 3.6 Å, respectively, with corresponding angles of 64.53° and 45.74°. In the S1 state, these distances changed to 6.5 Å and 3.7 Å, with angles of 36.26° and 35.99°, respectively. For BIMC-Protonated in the S0 state, the distances from O_1_ and O_2_ to the imidazole ring center were 5.0 Å and 3.7 Å, with angles of 64.82° and 43.63°. In the S1 state, these distances became 6.4 Å and 3.7 Å, with angles of 37.05° and 36.43°. The similarities in angles and key atomic distances between BIMC and BIMC-Protonated suggest that during this energy minimization process, the side chains of BIMC/BIMC-Protonated may have adopted different conformational isomers in S0 and S1-min states. Based on this observation, additional low-energy geometric conformers were selected for geometric optimization during the conformational search phase. The results, shown in Figure 8, present different conformational isomers of the side chain of BIMC-Protonated in the S1-min state. The conformations of BIMC in the S1-min state were highly similar to those of BIMC-Protonated. These four conformers showed no significant differences in their electronic spectra and energies. It can be concluded that the side chain remained stable during pH regulation and did not induce changes in local electronic structure. The pH-responsive properties of BIMC were found to primarily originate from the protonation/deprotonation of the imidazole ring.

**Figure 9:**
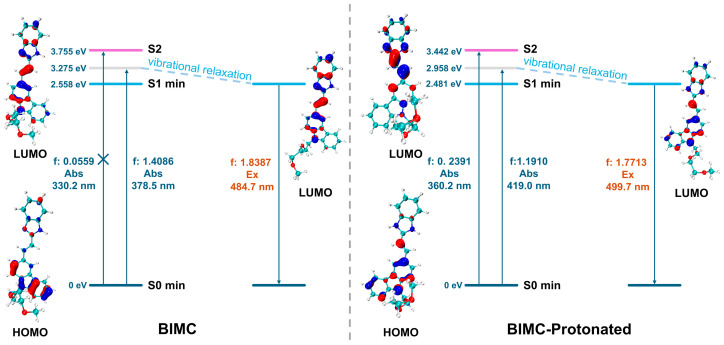
The electronic transition diagrams of BIMC and BIMC-Protonated. C: Cyan, N: blue, O: red, and H: white.

The spectral data results are listed in Table 2. The analysis shows that at this computational level, BIMC exhibited a calculated absorption wavelength of 378.5 nm and a fluorescence emission wavelength of 484.7 nm, suggesting that the emission energy was underestimated compared to the experimental value of 454.0 nm. The S0→S1 transition corresponded to electron excitation from the highest occupied molecular orbital (HOMO) to the lowest unoccupied molecular orbital (LUMO), contributing 98.37% with an oscillator strength of 1.4086. The S0→S2 transition primarily corresponded to the HOMO→LUMO+1 excitation, contributing 70.41%, followed by the HOMO-1→LUMO transition, contributing 18.99%, with an oscillator strength of 0.0559. This indicates that the S0→S1 transition represented the bright state, dominating the excitation process of BIMC. For BIMC-Protonated, the calculated absorption wavelength was 419.0 nm, and the fluorescence emission wavelength was 499.7 nm, suggesting that the emission energy was overestimated compared to the experimental value of 514.0 nm. The S0→S1 transition corresponded to the HOMO→LUMO excitation, contributing 98.62%. The S0→S2 transition primarily corresponded to the HOMO-1→LUMO excitation, contributing 93.49%, with a secondary contribution from the HOMO-1→LUMO+1 transition at 4.26%, showing an oscillator strength of 0.2391. The calculated energy diagram for excitation and emission processes is shown in Figure 9.

**Figure 10:**
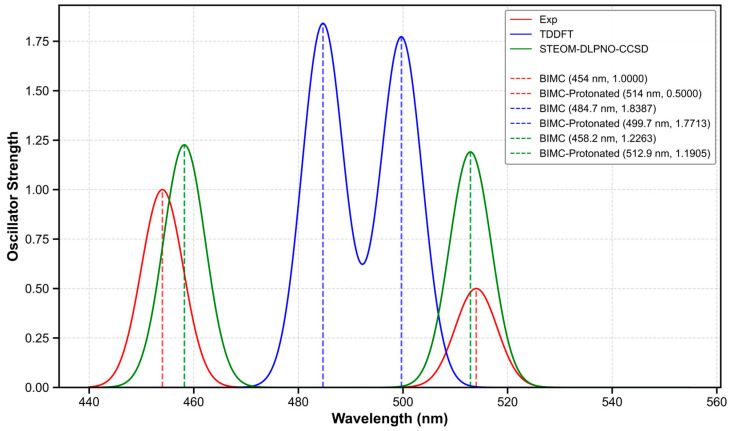
Experimental and theoretical spectra of BIMC and BIMC-Protonated.

**Figure 11:**
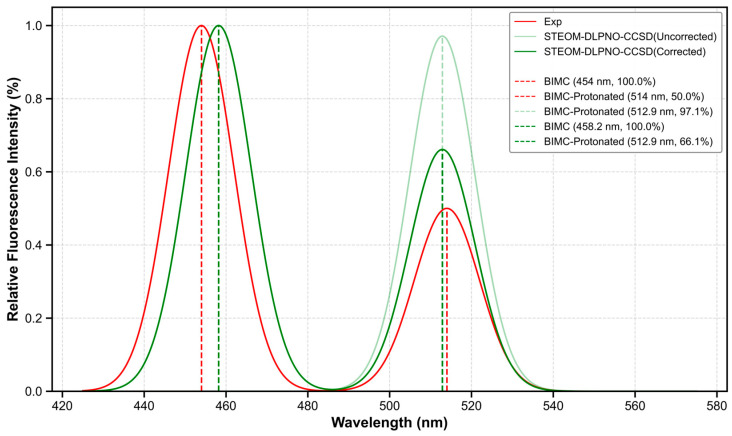
Comparison of experimental and theoretical spectra of BIMC and BIMC-Protonated after quantum yield correction.

To quantitatively compare the calculated and experimental spectra, electronic spectral calculations for BIMC and BIMC-Protonated were performed using the STEOM-DLPNO-CCSD method with the def2-TZVP basis set [14], based on the S1-min configuration. The spectral data are listed in Table 3. At this level of calculation, the computed fluorescence emission wavelength of BIMC was 458.2 nm with an oscillator strength of 1.2263, while BIMC-Protonated exhibited a computed fluorescence emission wavelength of 512.9 nm with an oscillator strength of 1.1905. At this computational level, the average error rate for fluorescence emission energy was merely 0.57%. These results are illustrated in Figure 10. To enhance the comparability between calculated and experimental spectra, we considered the influence of the fluorescence quantum yield. Under the approximation of neglecting intersystem crossing (ISC) processes, the calculated fluorescence rate for BIMC was K_r1_ = 2.94 × 10^8^ s^−1^ with an internal conversion rate of K_ic1_ = 4.31 × 10^7^ s^−1^, while BIMC-Protonated exhibited a fluorescence rate of K_r2_ = 2.28 × 10^8^ s^−1^ with an internal conversion rate of K_ic2_ = 1.26 × 10^8^ s^−1^. Subsequently, the calculated fluorescence quantum yields for BIMC and BIMC-Protonated were 87.0% and 64.4%, respectively. The corrected spectral intensities are presented in Table 4. Detailed computational procedures are described in Section 3. As shown in Figure 11, the estimation through fluorescence quantum yield significantly improved the accuracy of spectral calculations.

## 3. Materials and Methods

### 3.1. Experimental Materials

2,6-Bis(2-benzimidazolyl)pyridine (BBP, 98%) was purchased from Macklin Biochemical Co., Ltd. (Shanghai, China). Dimethyl sulfoxide (DMSO), hydrochloric acid (HCl, 1 mol/L), and sodium hydroxide (NaOH, 1 mol/L) were of analytical grade. The BBP stock solution (0.8 mol/L) was prepared by dissolving BBP in DMSO with the assistance of ultrasonication. The fluorescence measurements were performed on an FLS980 steady-state and transient fluorescence spectrometer (Edinburgh Instruments Ltd., Livingston, UK) with an excitation wavelength of 280 nm.

### 3.2. pK_a_ Prediction

The pK_a_ values of the target molecules were predicted by inputting SMILES representations into the Graph-pK_a_ model [19] via its web server. All predictions were conducted using the default settings provided by the server.

### 3.3. Molecular Dynamics

MD simulations were performed using the GROMACS 2020.6 package [20]. The force field parameters for BBP molecules were generated using Sobtop 1.0 (dev5) [21] based on the general AMBER force field (GAFF) [22]. Ten BBP/BBP-protonated molecules were randomly distributed in a periodic cubic simulation box with an edge length of 5 nm. The remaining space in the box was filled with DMSO solvent molecules, and Cl^−^ ions were added to neutralize the system. First, energy minimization was conducted using the steepest descent method for 50,000 steps. Subsequently, a 100 ps equilibration was performed in the NVT ensemble at 300 K, employing the velocity-rescaling method for temperature control with a coupling time constant of 0.1 ps. Subsequently, a 100 ps equilibration in the NPT was ensembled at 1.0 bar using the Berendsen barostat with a pressure coupling time constant of 3.0 ps and an isothermal compressibility of 4.5 × 10^−5^ bar^−1^. The production phase simulation was extended to 50 ns, utilizing the Nosé–Hoover thermostat and Parrinello–Rahman barostat. During the simulation, all bonds involving hydrogen atoms were constrained using the LINCS algorithm. Electrostatic interactions were treated using the particle mesh Ewald (PME) method with a fourth-order interpolation and a grid spacing of 0.10 nm. Both van der Waals and Coulomb interactions were truncated at 1.4 nm using the Verlet cut-off scheme with dispersion correction applied. System configurations were saved every 5 ps during the trajectory.

### 3.4. Quantum Chemical Computational Analysis 

Initial molecular spatial conformations were generated from SMILES strings using RDKit [23] and Open Babel [24]. To obtain energetically optimal conformers, conformational searches were conducted through simulated annealing using AmberTools23 [25]. During the simulated annealing process, the system temperature oscillated periodically between 1000 K and 0 K, with a time step of 2 fs and a total simulation time of 1 ns. The simulation employed a Langevin thermostat for temperature control, in the absence of periodic boundary conditions, and a cut-off radius of 12.0 Å. One hundred conformational snapshots were extracted at 0 K from the annealing trajectory, followed by single-point energy calculations at the PM6-DH+ [11] level using the MOPAC package [26] to identify the lowest-energy conformer.

All quantum chemical calculations were performed using the ORCA 5.0.4 program package [27]. First, ground-state geometry optimization and frequency calculations were conducted at the r2SCAN-3c [12] level. Based on the optimized ground-state geometry, excited-state geometry optimizations and frequency calculations for the first ten excited states were performed using non-adiabatic time-dependent density functional theory (TDDFT) with the PBE0 [13] functional, employing the def2-SV(P) [14] basis set, def2/J [28] auxiliary basis set, and RIJCOSX [29] approximation. TDDFT single-point energy calculations were performed on the PBE0 optimized geometries at the same level of theory. Additionally, the first five excited states were computed using the STEOM-DLPNO-CCSD method with the def2-TZVP [14] basis set and the def2/J and def2/JK [30] auxiliary basis sets. All calculations incorporated solvent effects using the SMD solvation model [31] for water and employed the defgrid3 grid for enhanced accuracy. All geometry optimizations were confirmed as energy minima through frequency calculations.

STEOM-DLPNO-CCSD computations were performed on a high-performance computing cluster equipped with dual AMD EPYC 7B12 processors, 512 GB DDR4 ECC memory operating at 2666 MT/s, and dual 4 TB storage units under CentOS 7.9.2009 environment. The remaining calculations were executed on a workstation featuring an Intel Core i7-10875H processor and 16 GB DDR4 memory at 3200 MT/s running Windows 11 23H2.

To achieve accurate predictions of fluorescence spectra, we developed a systematic correction method based on empirical relationships. Assuming that the non-radiative decay rate constant *k_nr_* is dominated by internal conversion (IC), we established an approximate expression for the fluorescence quantum yield φ.

(1) $$\varphi =\frac{k_{r}}{k_{r}+k_{ic}}$$

where *k_r_* represents the radiative transition rate constant and *k_ic_* represents the internal conversion rate constant. Through Einstein’s spontaneous emission formula [32], combined with the relationship between absorption and emission oscillator strengths (f_12_ = 1/3f_21_), and considering that the S1→S0 maintains constant level degeneracy, we could derive an approximate expression for the radiative transition rate constant *k_r_*.

(2) $$K_{r}=\frac{f_{21}\left(\omega_{21}^{2}e^{2}\right)}{2\pi \epsilon_{0}mc^{3}}$$

The internal conversion rate constant *k_ic_* was estimated using a widely adopted empirical formula [33]:

(3) $$k_{IC}\approx 10^{12-2\Delta E/\left(hc\right)}s^{-1}$$

Using *k_r_* and *k_ic_* obtained from these empirical models, we could estimate the theoretical fluorescence quantum yield φ through Equation (1). To better simulate experimentally observable quantities, the theoretical spectral intensities were corrected. The relationship between the corrected spectral intensity *I_B_* and the original intensity *I_A_* can be expressed as the following:

(4) $$\begin{array}{c} I_{B}=I_{A}\cdot \frac{\phi_{B}}{\phi_{A}}\cdot \frac{\lambda_{A}}{\lambda_{B}}\; \end{array}$$

To enhance computational efficiency and improve the accessibility of these quantum chemistry calculation software packages, we developed the EasySpecCalc program [34]. This program features an intuitive graphical user interface that streamlines the computational workflow. The streamlined workflow simplifies quantum chemical calculations, enabling experimental researchers to perform spectral predictions without requiring in-depth knowledge of the underlying processes. The program is freely available as open-source software on GitHub (https://github.com/CoomassieBrilliantBlue/EasySpecCalc, accessed on 20 November 2024).

The electrostatic potential (ESP) (Figure 5), frontier molecular orbitals (FMOs) (Figure 9), and average local ionization energy (ALIE) data were analyzed and visualized using Multiwfn 3.8 software [35,36].

## 4. Conclusions

This study employed quantum chemical calculations and molecular dynamics simulations to explore the photophysical properties of BBP and BIMC. For BBP, time-dependent density functional theory (TDDFT) calculations accurately predicted the fluorescence spectral characteristics under various protonation states, demonstrating excellent agreement with experimental results. Molecular dynamics simulations indicated that, in acidic environments, enhanced Coulomb interactions among BBP molecules and their propensity to form dimers collectively contributed to the observed fluorescence quenching phenomenon. For BIMC, a combination of TDDFT and STEOM-DLPNO-CCSD methods achieved highly accurate excitation energy predictions, which closely matched experimental data. Electrostatic potential and average local ionization energy (ALIE) analyses confirmed that the pH-responsive mechanism of BIMC is primarily driven by the protonation or deprotonation of its imidazole ring, while conformational changes in the side chains have minimal impact on its spectral properties. Using Einstein’s spontaneous emission formula and empirical formulas for internal conversion rates, the fluorescence rate, internal conversion rate, and fluorescence quantum yield of BIMC and its protonated forms were successfully calculated, yielding results consistent with experimental observations. These findings provide key molecular-level insights for designing pH-responsive fluorescent probes.

To facilitate the application of quantum chemical calculations in fluorescent probe development, we developed EasySpecCalc, a graphical user interface software that was made open-source on GitHub. This tool offers a convenient computational platform for future theoretical research and design iterations of fluorescent probes.

## Figures and Tables

**Table 1:** Theoretical and experimental electronic transition data for BBP and its protonated and deprotonated forms.

	Type	Electronic Transition	Wavelength (nm)	f
BBP	Absorption	S_0_→S_1_	320.6	0.8519
	Absorption	S_0_→S_2_	303.4	0.2940
	Absorption	S_0_→S_3_	290.0	0.3044
	Emission	S_1_→S_0_	369.1	1.2774
	Emission-Exp	S_1_→S_0_	385.0	/
BBP-Protonated	Absorption	S_0_→S_1_	324.5	0.8417
	Absorption	S_0_→S_2_	302.9	0.3461
	Emission	S_1_→S_0_	372.3	1.1886
	Emission-Exp	S_1_→S_0_	396.0	/
BBP-Deprotonated	Absorption	S_0_→S_1_	328.9	0.8545
	Absorption	S_0_→S_2_	315.0	0.1571
	Emission	S_1_→S_0_	381.6	1.2980
	Emission-Exp	S_1_→S_0_	410.0	/

**Table 2:** Theoretical (under the TDDFT method) and experimental electronic transition data for BIMC and its protonated forms.

	Type	Electronic Transition	Wavelength (nm)	f
BIMC	Absorption	S_0_→S_1_	378.5	1.4086
	Absorption	S_0_→S_2_	330.2	0.0559
	Emission	S_1_→S_0_	484.7	1.8387
	Emission-Exp	S_1_→S_0_	454.0	/
BIMC-Protonated	Absorption	S_0_→S_1_	419.0	1.1910
	Absorption	S_0_→S_2_	360.2	0.2391
	Emission	S_1_→S_0_	499.7	1.7713
	Emission-Exp	S_1_→S_0_	514.0	/

**Table 3:** Theoretical (under the STEOM-DLPNO-CCSD method) and experimental electronic transition data for BIMC and its protonated forms.

	Type	Electronic Transition	Wavelength (nm)	f
BIMC	Emission	S_1_→S_0_	458.2	1.2263
	Emission-Exp	S_1_→S_0_	454.0	/
BIMC-Protonated	Emission	S_1_→S_0_	512.9	1.1905
	Emission-Exp	S_1_→S_0_	514.0	/

**Table 4:** Comparative analysis of normalized spectral intensities for BIMC and BIMC-Protonated using different estimation methods.

	Osc. Strength	Fl. Quantum Yield	Experimental
BIMC *	100%	100%	100%
BIMC-Protonated	97.1%	66.1%	50%

* All values are normalized to the intensity of BIMC (100%).

## Data Availability

The authors confirm that the data supporting the findings of this study are available within the article.
